# Comparison of Performance, Egg Quality, and Egg Cost of Different Laying Genotypes in Free-Range System from 21 to 44 Weeks of Age

**DOI:** 10.3390/ani15010086

**Published:** 2025-01-02

**Authors:** Ali Aygun, Doğan Narinç, Hasan Arısoy

**Affiliations:** 1 Department of Animal Science, Agriculture Faculty, Selçuk University, Konya 42130, Turkey; 2 Department of Animal Science, Agriculture Faculty, Akdeniz University, Antalya 07070, Turkey; narincd@gmail.com; 3 Department of Agricultural Economics, Agriculture Faculty, Selçuk University, Konya 42130, Turkey; arisoy@selcuk.edu.tr

**Keywords:** Lohmann Sandy, Lohmann Brown, ATAK-S, egg cost, performance

## Abstract

The aim of this study was to compare the performance, egg quality and economic aspects of laying hybrids of different genotypes in free-range system. In the study, three different laying genotypes (Lohmann Brown, Lohmann Sandy and ATAK-S genotype) were used. Each group consisted of four replicates and each replicate contained 20 hens. Egg production, broken–cracked egg ratio, egg weight, feed consumption, feed efficiency, and viability were determined as performance traits. Eggshell strength, Haugh unit, yolk color, and shell color were determined as egg quality traits. The costs of eggs were assessed for economic analysis. The Lohmann Sandy genotype produced more eggs than the Lohmann Brown genotype (*p* < 0.05). The ATAK-S genotype reached 50% egg production age sooner than other genotypes (*p* < 0.05). The Lohmann Sandy and Lohmann Brown genotypes produced fewer broken eggs and heavier eggs compared to the ATAK-S genotype (*p* < 0.05). The ATAK-S genotype consumed more feed than the Lohmann Sandy and Lohmann Brown genotypes (*p* < 0.05). The Lohmann Sandy and Lohmann Brown genotypes have better feed efficiency compared to the ATAK-S genotype (*p* < 0.05). Eggs from the Lohmann Sandy genotype exhibit greater resistance to breakage compared to those from other genotypes (*p* < 0.05). The highest quality of albumen was identified in the eggs of the Lohmann Brown genotype (*p* < 0.05). The yolk color of Lohmann Brown genotype eggs is darker than those of other genotypes (*p* < 0.05). The shell color of Lohmann genotype eggs is a darker brown compared to eggs of other genotypes (*p* < 0.05). The cheapest egg cost was determined from eggs produced from the Lohmann Sandy genotype (*p* < 0.05). According to the results obtained from our study, it would be more appropriate to prefer the Lohmann Sandy genotype in the free-range system.

## Simple Summary

In recent years, customers have increasingly favored eggs from cage-free systems, believing them to be healthier and more flavorful. It has become increasingly necessary to evaluate the genotypes raised in these systems in terms of production, quality, and economic viability. The aim of this study was to compare the performance, egg quality and economic aspects of Lohmann Sandy, Lohmann Brown and ATAK-S genotypes reared in a free-range system. In terms of performance and egg quality, the Lohmann Sandy and Lohmann Brown genotypes are better than the ATAK-S genotype. The ATAK-S genotype is better than other genotypes in terms of 50% egg production age. The difference between genotypes in terms of survival rate was statistically insignificant. We determined that the Lohmann Sandy genotype eggs had the lowest price.

## 1. Introduction

Animal rights advocates have pressed for a ban on traditional cage systems, claiming that chickens raised in traditional cages are unable to exhibit natural behaviors. They have advocated the use of alternative production systems that allow the birds to exhibit natural behavior while also protecting animal welfare. As an alternative to the traditional cage system for laying hens, enriched cages, aviaries, barns, and free-range systems are used [1,2,3,4]. Consumers believe that eggs produced in an alternative system are healthier, more natural, and tastier than traditional cage eggs [5,6,7,8]. Field tests in the target housing system of layer genotypes to be used in egg production in free-range systems has become increasingly important. White and brown layer genotypes are generally used in egg production in free-range systems. Brown layers are mostly used in free-range systems around the world [9,10,11,12]. Dikmen et al. [13] found that the Lohmann Brown genotype’s hen-day egg production was 89.3%, the egg weight was 59.8 g, the feed consumption was 124.6 g/day, the feed efficiency was 2.17 g feed/g egg, and the broken egg rate was 0.35 in a free-range environment. Tutkun et al. [14] determined the hen-day egg production of the Lohmann Brown genotype as 78.2%, daily feed consumption as 111.4 g, feed efficiency as 2.45 g feed/g egg, egg weight as 58.8 g, and broken egg rate as 1.11% in the free-range system. The ATAK-S genotype, a local layer hybrid, has increasingly been used in the rural areas of Turkey [14,15,16,17]. The ATAK-S layer hybrid has a 50% production age of 144 days, egg production up to 72 weeks of age is 314, and average feed intake throughout the laying phase is 115–118 g/day [18]. Türker et al. [15] conducted a study in a free-range system using the ATAK-S genotype, determining egg production at 78.4%, egg weight at 63.11 g, feed consumption at 130.1 g/day, feed evaluation at 2.53 g feed/g egg, and 50% egg production age at 160 days. Sözcü et al. [16] stated that the hen-day egg production of the ATAK-S genotype was 74.9%, egg weight was 62.9 g, feed consumption was 117.2 g/day, feed efficiency was 2.54 g feed/g egg, and the broken egg rate was 4.6% in the free-range system. In recent years, egg-laying genotypes with cream shell color and mostly used in free-range systems have been improved. The genotypes are commercially developed by Lohmann as Sandy and Silver, by Hy-Line as Pink and Sonia, and by H&N International as Coral. The body weight values of the Lohmann Sandy genotype are 1350 g at 17 weeks, the age at which 50% yield is reached is 140–150 days, the average daily feed consumption varies between 105 and 118 g, and the feed efficiency is between 2.00 and 2.15 [19]. In Turkey, the Lohmann Brown and ATAK-S genotypes were mostly used in the comparison of genotypes in the free-range system. According to our literature research, there have been few studies conducted with Lohmann Sandy in the free-range system [20]. Alkan [20] determined the egg quality characteristics of the Lohman Sandy genotype in the free-range system. Identifying genotypes suitable for rearing in a free-range system based on performance, egg quality, and cost is essential for egg producers. For this reason, the purpose of this study was to investigate the most popular layer genotypes in our region’s free-range system in terms of performance, egg quality, and egg cost.

## 2. Materials and Methods

This research was carried out in accordance with the laws and regulations of the Ministry of Agriculture of the Republic of Turkey on animal management and welfare (22 November 2014; Number: 29183). In addition, the study fully complied with the SCAHAW (2003) recommendations for the free-range alternative layer rearing system. The experiment was conducted at the poultry facility of the Department of Animal Sciences at the University of Selçuk in Turkey. Three different genotypes, commercial (Lohmann Sandy, (LS); Lohmann Brown, LB) and domestic (ATAK-S, A), were used in the study. The experiment consisted of 3 genotype groups, and each group consisted of 4 replications. A total of 240 fifteen-week-old laying hens were randomly allocated to 3 treatments of 80 hens each, with 4 replicates per treatment.

Twelve individual poultry pens were used in the study. Each poultry pen measureed 2.0 × 1.5 × 2.0 m (length × width × height) and was made of a sandwich panel. A 180 cm high wire fence surrounded the outdoor areas, and a net covered them. A circular feeder with a diameter of 30 cm and a nipple drinker for every 10 animals were placed in each poultry pens. The multi-storey perches of 15 cm per hen were provided separately in the indoor and outdoor areas. Wood shavings were used as litter material in the indoor area for the hens. A nest box measuring 140 × 40 × 40 cm was used in each poultry pen. Animals were allowed access to the outdoor area for a minimum of 8 h daily. The stocking density in the indoor area was 6 hens/m^2^, while the outdoor area provided 4 m^2^ per hen. At 15 weeks of age, the chickens obtained 10 h of light daily, which gradually increased to 16 h per day by adding 30 min each week. Feed and water were given ad libitum. The animals were given developer feed (15.5% Crude Protein (CP), 2750 kg Kcal/Metabolizable energy (ME), 1% calcium, 0.40% available P in the 15th–18th weeks, pre-layer feed (17.5% CP, 2750 kg Kcal/ME, 2% calcium, 0.45% available P) until 5% egg production, and then first-period egg feed (18% CP, 2750 kg Kcal/ME, 4% calcium, 0.45% available P). The outdoor area of the free-range chicken pens was planted with clover-perennial grass. In the study, data were collected between 15 and 44 weeks of age. We used sunshades in our research to minimize the effects of daylight temperature on the hens. The optimum temperature range for hens is specified for the indoor area.

Egg production in the treatment groups was recorded on a daily basis beginning with the experiment. These data were utilized for calculating hen-day egg production as well as 5% and 50% egg production age parameters. To determine the egg weight, all eggs in each poultry pen were weighed for two consecutive days every two weeks. The number of broken-cracked eggs was determined daily. The feed efficiency was calculated by determining feed consumption during four-week periods. Chicken deaths were recorded daily. Livability was determined by using these data.

For egg quality analyses, 5 eggs were randomly taken from the eggs produced on 2 consecutive days (the subgroup level) in the last week of each 4-week period. Egg quality characteristics, including eggshell color, eggshell breaking strength, Haugh unit, and yolk color, were examined. The blunt end of the egg was measured with a color device (Konica Minolta, CR-400, Japan). The brightness–darkness (L*) color value was determined according to the CIELab color system. Before the analysis measurements, the device was calibrated using the reference plate (L* = 97.10, a* = −4.88, b* = 7.04) [21]. Eggshell breaking strength was determined with the device (ERTEST, Ankara, Turkey). Egg albumen height was measured with the height gauge device (Tresna, Essen, Germany). The Haugh unit was then calculated using egg white height and egg weight [22]. Egg yolk color was measured with a portable colorimeter device (Konica Minolta, CR-400, Chiyoda, Japan). The research calculated the cost of eggs for each unit. The approach for calculating combined costs, as outlined in Equation (1), was used for determining the cost of eggs [23].

(1) $$Egg\;cost=\frac{Total\;production\;cost-waste\;product\;income}{productionamount}$$

One-way analysis of variance (one-way ANOVA) was used in data analysis. A multiple comparison test (Tukey’s test) was used in comparisons between groups. All hypothesis tests were performed at a significance level of 0.05, and the Minitab 16 package program was used for statistical analysis.

## 3. Results

**Figure 1:**
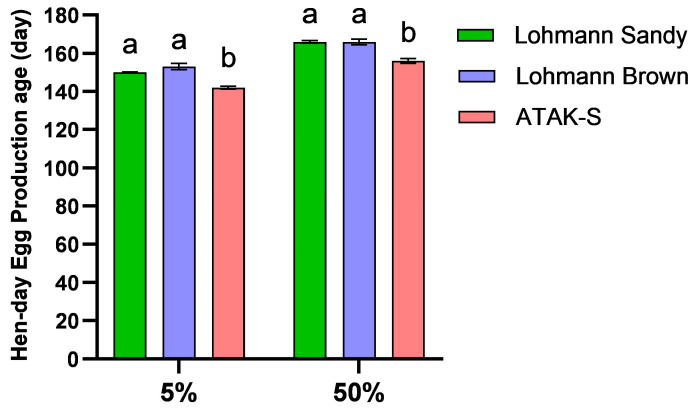
The effects of genotype on 5% and 50% egg production age. ^a,b^ The difference between the groups indicated by different letters is statistically significant (*p* < 0.05).

The effect of genotype on 5% and 50% egg production ages was significant (Figure 1). The age at which genotype A produces 5% egg production (142 days) is lower than that of genotype LS (150 days) and genotype LB (153 days) (*p* < 0.05). However, the difference in egg production age at 5% between the LS genotype and the LB genotype was found to be statistically insignificant (*p* > 0.05). The age at which genotype A produces 50% egg production (157 days) is lower than that of genotype LS (166 days) and genotype LB (167 days) (*p* < 0.05). However, the difference in egg production age at 50% between the LS genotype and the LB genotype was found to be statistically insignificant (*p* > 0.05).

The effect of genotype on hen-day egg production (%) was insignificant between 29 and 32 weeks, 33 and 36 weeks, and 41 and 44 weeks (*p* > 0.05), while egg production was statistically significant at 21–24 weeks, 25–28 weeks, and 37–40 weeks (*p* < 0.05) (Table 1). Throughout all periods (21–44 weeks), egg production from the LS genotype (80.69%) was higher than that from the LB genotype (71.43%) (*p* < 0.05), but similar to that from the A genotype (78.39%) (*p* > 0.05). There was no statistically significant difference in egg production between the A genotype (78.39%) and the LB genotype (71.43%) (*p* > 0.05).

The statistically significant differences in broken-cracked eggs among the groups were observed during all periods (*p* < 0.05) (Table 2). The highest broken-cracked egg rate in all periods was detected in the A genotype (*p* < 0.05), and it was observed that there was a statistically insignificant difference between the LS genotype and the LB genotype in terms of broken-cracked egg rate (*p* > 0.05).

Statistically significant differences in egg weights among the groups were noted throughout all periods (*p* < 0.05) (Table 3). The A genotype exhibited the lowest egg weight throughout all periods, while the difference in egg weight between the LS genotype and the LB genotype was found to be statistically insignificant.

The effect of genotype on feed consumption was significant during any period except for weeks 37–40 and 41–44 (*p* < 0.05) (Table 4). The highest feed consumption during the trial was determined in genotype A (*p* < 0.05). However, the difference between the LS genotype and the LB genotype in terms of feed consumption was statistically insignificant (*p* > 0.05).

The effect of genotype on feed efficiency was significant throughout all periods except for weeks 37–40 and 41–44 (*p* < 0.05) (Table 5). The worst feed efficiency value during the trial was determined in genotype A (*p* < 0.05). The difference in feed efficiency between the LS genotype and the LB genotype was statistically insignificant (*p* > 0.05).

The livability was assessed at 100% for the LS group, 86.25% for the LB group, and 95% for the A group, with no statistically significant difference between the groups (*p* > 0.05) (data not presented).

The effect of genotype on eggshell strength was significant throughout all periods (*p* < 0.05) (Table 6). The LS genotype exhibited the strongest eggshell strength, whereas the A genotype had the lowest eggshell strength (*p* < 0.05).

The effect of genotype on the Haugh unit was significant throughout all periods (*p* < 0.05) (Table 7). Overall analysis revealed that the A genotype had the lowest Haugh unit value and the LB genotype had the greatest Haugh unit value (*p* < 0.05).

**Figure 2:**
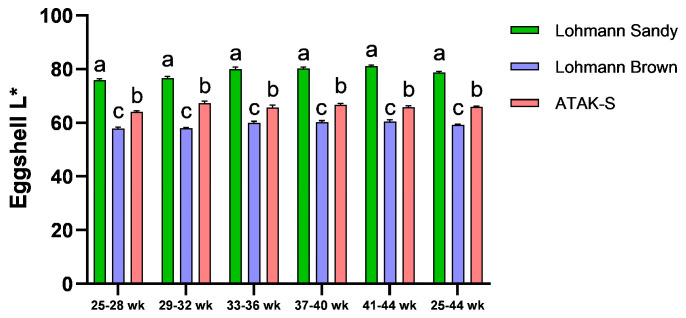
The effects of genotype on eggshell L* value. ^a–c^ The difference between the groups indicated by different letters is statistically significant (*p* < 0.05).

The effect of genotype on eggshell L* value was significant throughout all periods (*p* < 0.05) (Figure 2). In all periods, the highest eggshell L* value was determined in the LS genotype, and the lowest shell L* value was determined in the LB genotype (*p* < 0.05).

**Figure 3:**
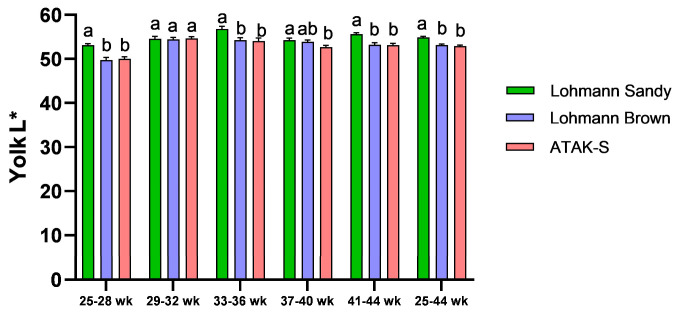
The effects of genotypes on yolk L* value. ^a,b^ The difference between the groups indicated by different letters is statistically significant (*p* < 0.05).

The effect of genotype on yolk L* value was significant throughout all periods except for weeks 29–32 (*p* < 0.05) (Figure 3). In general, the highest yolk L* value was determined in the LS genotype (*p* < 0.05). However, the difference between the LB genotype and the A genotype in terms of yolk L* was statistically insignificant (*p* > 0.05).

**Figure 4:**
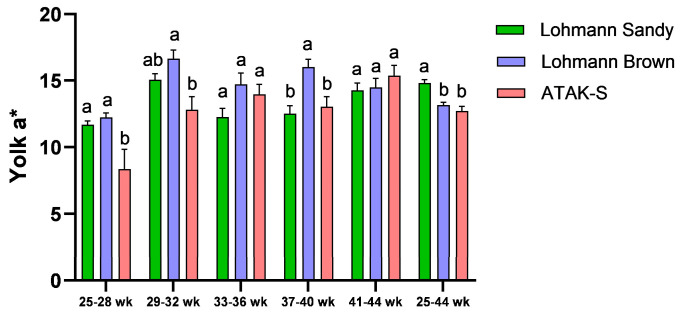
The effects of genotype on yolk a* value. ^a,b^ The difference between the groups indicated by different letters is statistically significant (*p* < 0.05).

The effect of genotype on yolk a* value was significant throughout all periods except for weeks 33–36 and 41–44 (*p* < 0.05) (Figure 4). During the trial period (25–44 weeks), it was observed that the yolk a* value of the LB genotype was higher than the yolk a* value of the LS genotype and A genotype (*p* < 0.05). The difference in yolk a* value between the LS genotype and the A genotype was statistically insignificant (*p* > 0.05).

**Figure 5:**
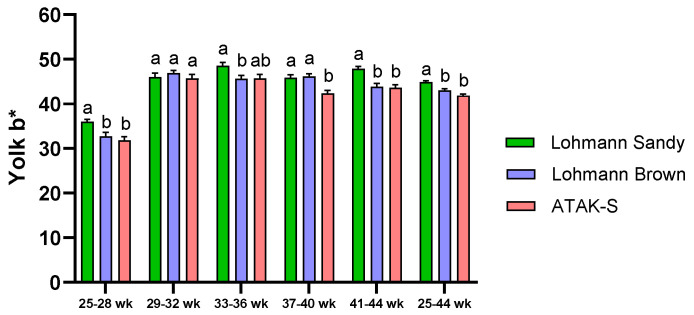
The effects of genotype on yolk b* value. ^a,b^ The difference between the groups indicated by different letters is statistically significant (*p* < 0.05).

The effect of genotype on yolk b* value was significant throughout all periods except for weeks 29–32 (*p* < 0.05) (Figure 5). During the trial period (25–44 weeks), it was observed that the yolk b* value of the LS genotype was higher than the yolk b* value of the LB genotype and A genotype (*p* < 0.05). The difference in yolk b* value between the LB genotype and the A genotype was statistically insignificant (*p* > 0.05).

**Figure 6:**
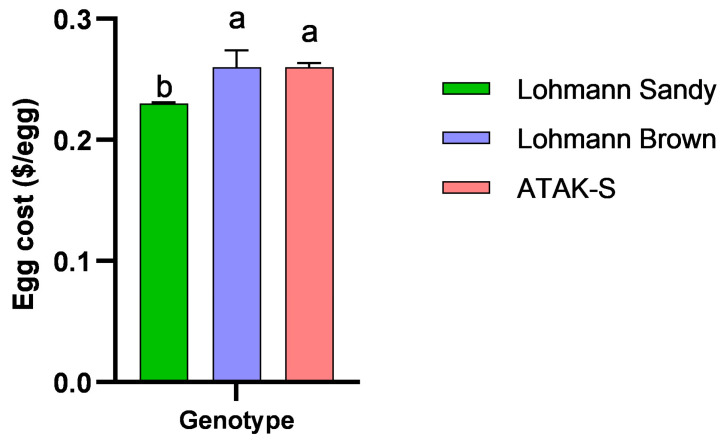
The effects of genotypes on egg cost. ^a,b^ The difference between the groups indicated by different letters is statistically significant (*p* < 0.05).

The effect of genotype on egg cost was significant (*p* < 0.05) (Figure 6). The lowest egg cost was determined in the LS genotype (0.23 USD/egg) (*p* < 0.05). The difference in egg cost between the LB genotype (0.26 USD/egg) and the A genotype (0.26 USD/egg) was statistically insignificant (*p* > 0.05).

## 4. Discussion

In our study, the ATAK-S genotype reached both 5% and 50% yield age earlier. Similar results are also observed in hybrid catalog values. The ATAK-S genotype has a 50% yield age of 144 days [18], while both the Lohmann Brown and Lohmann Sandy genotypes have a 50% yield age of 145 days, according to their respective catalog data [19,24]. Sekeroglu et al. [25] established the 50% yield age of the ATAK-S genotype as 160 days in a free-range system. Similarly, Dikmen et al. [13] determined the 50% yield age of the Lohman Brown genotype as 160 days in the free-range system. The age at 50% egg production can be influenced by a number of factors, including chick weight, the environment throughout the raising period, and the age at sexual maturity [26,27]. The Lohmann Sandy genotype is recognized as the most efficient in hen-day egg production. No significant difference was found in egg production between the Lohmann Brown and Atak-S genotypes. According to several researchers, in a free-range system, the Lohmann brown genotype produces more eggs than the Atak-S genotype [28,29]. The reason for these differences is that egg production can be affected by many factors. These factors include age at sexual maturity, body weight at sexual maturity, feeding, lighting, and diseases [27,30].

The ATAK-S genotype produced a greater number of broken-cracked eggs compared to the Lohmann Sandy and Lohmann Brown genotypes. Kop-Bozbay et al. [31] documented a broken-cracked rate of 2.90% for eggs produced from the Lohmann Brown genotype in the free-range system. Aygun et al. [4] reported that the rate of broken-cracked eggs from the ATAK-S genotype in the free-range system ranged from 1.78% to 4.91%. Approximately 8–10% of eggs produced in egg-producing enterprises are cracked, resulting in economic losses [32]. Some researchers have indicated that genotype influences the incidence of broken or cracked eggs in free-range systems [16,33]. The higher rate of broken-cracked eggs in the free-range system may result from the increased motor activity of the animals. Moreover, nutrient deficiency, high temperature, and stress may influence the incidence of broken eggs [32,34]. The genotypes of laying hens differ in their ability to tolerate heat stress [35,36]. The ATAK-S genotype produced lower egg weights compared to Lohmann Sandy and Lohmann Brown. Egg weight is an important evaluation characteristic for consumers. All genotypes produced eggs within the medium weight category (53–63 g), representing the optimal egg weight. In the free-range system, the Lohmann Sandy genotype’s egg weight was found to be 58.84 g by Alkan [20] and 59.9 g by Akyol and Denli [37]. The factors that influence egg weight include genotype, health status, hen age, body weight, and hen diet [38,39,40].

The ATAK-S genotype consumed more feed than the Lohmann Sandy and Lohmann Brown genotypes. Baldinger and Bussemas [41] reported the average feed consumption of the Lohmann Sandy genotype in the organic system as 143 g. Sözcü et al. [29] determined that the feed consumption of the Lohmann Brown and ATAK-S genotypes in the free-range system was 130.9 g/day and 122.8 g/day, respectively. The difference in feed consumption between genotypes may be due to differences in body weight, motor activity, stress, and the consumption of forage material in the outdoor area [42,43,44]. Blair [45] stated that the utilization of pasture material might decrease feed consumption by up to 20%. The feed efficiency of the ATAK-S genotype is poorer than that of the Lohmann Sandy and Lohmann Brown genotypes. Feed efficiency is assessed using egg weight and feed consumption measures. The ATAK-S genotype exhibits lower egg weight and higher feed consumption than other genotypes, resulting in poorer feed efficiency. Consistent with studies showing that genotype has an important effect on feed efficiency [4,15,29]. Türker et al. [15] and Sözcü et al. [29] indicate that the feed efficiency of the ATAK-S genotype in the free-range system is inferior to that of foreign layer hybrids.

The Lohmann Sandy genotype exhibited the strongest eggshell strength, while the ATAK-S genotype demonstrated the lowest eggshell strength. The strength of eggshells is an important characteristic in the collection, transportation, and storage of eggs. Eggshell strength is a critical economic aspect, as cracked eggs are discarded, leading to financial losses for producers [46]. In the free-range system, the eggshell strength of the ATAK-S genotype is weaker than that of foreign layer hybrids [15,29]. The effect of genotype on eggshell strength is consistent with some studies that show it is important [47,48]. The highest Haugh unit was detected in Lohmann Brown eggs, and the lowest Haugh unit was detected in ATAK-S eggs. The Haugh unit is an important internal quality measure developed by the scientist Haugh in 1937, determined by the weight of the egg and the height of the egg white. A higher Haugh unit indicates better egg quality and extends the egg’s shelf life during storage. Our findings are consistent with previous research indicating that genotype influences the Haugh unit in the free-range system [16,48,49,50].

The highest L* value (brightness) was found in Lohmann sandy eggs, and the lowest L* value (darkness) was identified in Lohmann Brown eggs. Eggshell color preferences vary among consumers in different countries. Consumers evaluate eggshell color and color uniformity as egg quality. Consumers in Australia, the United States, Sweden, and Spain prefer white-shelled eggs, while consumers in China, France, and Portugal prefer brown-shelled eggs [51]. The findings from our research align with studies demonstrating that genotype significantly influences eggshell color [47,52,53]. The coloration of the eggshell is chiefly derived from pigment constituents, namely biliverdin and protoporphyrin IX. Brown eggshells result from protoporphyrin IX, whereas blue eggshells arise from biliverdin and protoporphyrin IX [54,55,56]. Darker eggshell colors exhibiting a brown color correlated with reduced L* values and increased accumulation of protoporphyrin IX [57,58].

The yolk color of Lohmann Sandy eggs was found to be brighter than that of Lohman Brown and ATAK-S genotype eggs. It was observed that the yolk color of Lohmann brown eggs was higher in redness than the yolk color of Lohman Sandy and ATAK-S genotype eggs. Narinç et al. [59] demonstrated in their research on the Roche yolk color scale that the a* value escalates with the intensity of yolk color. Lohmann Brown eggs possess a darker yolk color compared to Lohmann Sandy and ATAK-S genotype eggs.

The results obtained from our study are consistent with studies indicating that genotype significantly affects egg yolk color [16,60,61]. The intensity of yolk color is a significant characteristic for consumers, mostly determined by nutritional factors [62,63]. The carotenoids in a chicken’s diet mostly influence the color of its yolk. Nonetheless, variations in carotenoid concentrations in feed and their bioavailability from different sources can substantially influence egg yolk content [64]. The consumption of carotenoids in pasture results in yolks that are darker yellow–orange in color and have higher color scores [65].

The most economical egg cost (USD 0.23/egg) was identified in Lohmann Sandy eggs. The principal factor influencing egg costs is feed consumption. On chicken farms, feed expenses are roughly 70–75% of overall production costs [66,67]. Chang et al. [68] reported that eggs from free-range farms in the United States are priced at USD 0.43 each. Aygun et al. [4] established the egg cost at USD 0.11 per egg in their research involving the ATAK-S genotype in the free-range system.

## 5. Conclusions

According to our study results, genotype has a significant effect on performance, egg quality, and egg cost. The results obtained provide important results in terms of performance, egg quality, and egg cost of the genotypes to be used for egg production in the free-range system. Our investigation indicates that the Lohmann Sandy genotype could have advantages for egg production in free-range systems compared to other genotypes.

## Figures and Tables

**Table 1:** The effects of genotype on hen-day egg production (%).

Genotype	21–24 wk	25–28 wk	29–32 wk	33–36 wk	37–40 wk	41–44 wk	21–44 wk
LS	16.34 ^b^	89.24 ^a^	95.18	93.84	96.29 ^a^	93.27	80.69 ^a^
LB	5.13 ^c^	71.34 ^b^	90.49	89.50	84.32 ^b^	87.81	71.43 ^b^
A	39.96 ^a^	87.99 ^ab^	89.02	81.83	84.38 ^b^	87.13	78.39 ^ab^
SEM	2.05	4.25	2.75	3.50	2.83	3.00	2.20
*p* value	0.000	0.027	0.305	0.101	0.023	0.325	0.038

LS: Lohmann Sandy, LB: Lohmann Brown, A: ATAK-S, SEM: Standard error mean, ^a–c^ The difference between the groups indicated with different letters in the same column is significant (*p* < 0.05).

**Table 2:** The effects of genotypes on broken-cracked eggs (%).

Genotype	21–24 wk	25–28 wk	29–32 wk	33–36 wk	37–40 wk	41–44 wk	21–44 wk
LS	4.55 ^b^	1.86 ^b^	1.35 ^b^	0.85 ^c^	0.51 ^c^	0.89 ^b^	1.67 ^b^
LB	1.07 ^c^	1.86 ^b^	2.07 ^b^	2.65 ^b^	2.36 ^b^	2.05 ^b^	2.01 ^b^
A	8.36 ^a^	6.12 ^a^	7.60 ^a^	8.09 ^a^	6.80 ^a^	7.84 ^a^	7.46 ^a^
SEM	0.72	0.41	0.54	0.41	0.35	0.53	0.33
*p* value	0.000	0.000	0.000	0.000	0.000	0.000	0.000

LS: Lohmann Sandy, LB: Lohmann Brown, A: ATAK-S, SEM: standard error mean, ^a–c^ The difference between the groups indicated with different letters in the same column is significant (*p* < 0.05).

**Table 3:** The effects of genotypes on egg weight (g).

Genotype	21–24 wk	25–28 wk	29–32 wk	33–36 wk	37–40 wk	41–44 wk	25–44 wk
LS	-	55.52 ^a^	59.78 ^a^	59.72 ^a^	62.56 ^a^	63.41 ^a^	60.20 ^a^
LB	-	56.46 ^a^	59.09 ^a^	59.31 ^a^	61.90 ^a^	64.02 ^a^	60.15 ^a^
A	-	52.24 ^b^	54.89 ^b^	54.42 ^a^	57.23 ^b^	58.04 ^b^	55.37 ^b^
SEM	-	0.71	0.41	0.61	0.55	0.63	0.49
*p* value	-	0.006	0.000	0.000	0.000	0.000	0.000

LS: Lohmann Sandy, LB: Lohmann Brown, A: ATAK-S, SEM: standard error mean, ^a,b^ The difference between the groups indicated with different letters in the same column is significant (*p* < 0.05).

**Table 4:** The effects of genotypes on feed consumption (g/hen/day).

Genotype	21–24 wk	25–28 wk	29–32 wk	33–36 wk	37–40 wk	41–44 wk	21–44 wk
LS	78.79 ^c^	100.73 ^b^	102.6 ^b^	97.6 ^b^	104.9	109.2	98.9 ^b^
LB	87.90 ^b^	101.98 ^b^	100.3 ^b^	105.5 ^ab^	105.5	103.3	100.8 ^b^
A	103.99 ^a^	120.51 ^a^	115.2 ^a^	114.9 ^a^	108.3	108.2	111.8 ^a^
SEM	2.27	1.94	2.56	4.11	3.73	4.25	2.32
*p* value	0.000	0.000	0.006	0.045	0.803	0.598	0.007

LS: Lohmann Sandy, LB: Lohmann Brown, A: ATAK-S, SEM: standard error mean, ^a–c^ The difference between the groups indicated with different letters in the same column is significant (*p* < 0.05).

**Table 5:** The effects of genotypes on feed efficiency (g feed/g egg).

Genotype	21–24 wk	25–28 wk	29–32 wk	33–36 wk	37–40 wk	41–44 wk	25–44 wk
LS	-	1.81 ^b^	1.72 ^b^	1.63 ^b^	1.68	1.72	1.71 ^b^
LB	-	1.81 ^b^	1.70 ^b^	1.78 ^b^	1.71	1.61	1.72 ^b^
A	-	2.31 ^a^	2.10 ^a^	2.11 ^a^	1.89	2.86	2.06 ^a^
SEM	-	0.04	0.05	0.08	0.06	0.06	0.05
*p* value	-	0.000	0.000	0.006	0.104	0.059	0.001

LS: Lohmann Sandy, LB: Lohmann Brown, A: ATAK-S, SEM: standard error mean, ^a,b^ The difference between the groups indicated with different letters in the same column is significant (*p* < 0.05).

**Table 6:** The effects of genotypes on eggshell strength (kg).

Genotype	21–24 wk	25–28 wk	29–32 wk	33–36 wk	37–40 wk	41–44 wk	25–44 wk
LS	-	4.878 ^a^	4.790 ^a^	4.915 ^a^	5.041 ^a^	4.865 ^a^	4.898 ^a^
LB	-	4.598 ^a^	4.367 ^b^	4.311 ^b^	4.470 ^b^	4.197 ^b^	4.389 ^b^
A	-	4.020 ^b^	3.748 ^c^	3.653 ^c^	3.725 ^c^	3.494 ^c^	3.728 ^c^
SEM	-	0.10	0.12	0.10	0.09	0.13	0.04
*p* value	-	0.000	0.000	0.000	0.000	0.000	0.000

LS: Lohmann Sandy, LB: Lohmann Brown, A: ATAK-S, SEM: standard error mean, ^a–c^ The difference between the groups indicated with different letters in the same column is significant (*p* < 0.05).

**Table 7:** The effects of genotypes on Haugh unit.

Genotype	21–24 wk	25–28 wk	29–32 wk	33–36 wk	37–40 wk	41–44 wk	25–44 wk
LS	-	89.16 ^ab^	87.47 ^b^	95.80 ^a^	86.17 ^b^	83.67 ^b^	87.14 ^b^
LB	-	92.50 ^a^	94.32 ^a^	89.21 ^b^	92.75 ^a^	89.44 ^a^	92.96 ^a^
A	-	85.40 ^b^	82.15 ^c^	83.90 ^c^	83.68 ^b^	81.45 ^b^	83.32 ^c^
SEM	-	1.18	0.93	1.44	1.28	1,09	0.59
*p* value	-	0.000	0.000	0.000	0.000	0.000	0.000

LS: Lohmann Sandy, LB: Lohmann Brown, A: ATAK-S, SEM: standard error mean, ^a–c^ The difference between the groups indicated with different letters in the same column is significant (*p* < 0.05).

## Data Availability

The data that support the findings of this study are available on request from the corresponding author.
